# Pathological profiles and clinical management challenges of breast cancer emerging in young women in Indonesia: a hospital-based study

**DOI:** 10.1186/s12905-019-0724-3

**Published:** 2019-02-06

**Authors:** Sumadi Lukman Anwar, Clarista Adelia Raharjo, Rahma Herviastuti, Ery Kus Dwianingsih, Didik Setyoheriyanto, Widya Surya Avanti, Lina Choridah, Wirsma Arif Harahap, Darwito, Teguh Aryandono, Wahyu Wulaningsih

**Affiliations:** 1 Division of Surgical Oncology-Department of Surgery, Faculty of Medicine, Public Health and Nursing, Universitas Gadjah Mada (Dr. Sardjito Hospital), Jalan Kesehatan No 1, Yogyakarta, 55281 Indonesia; 2 PILAR Research and Education, 20 Station Road, Cambridge, CB1 2JD UK; 3 Department of Anatomical Pathology - Faculty of Medicine, Public Health and Nursing, Universitas Gadjah Mada (Dr. Sardjito Hospital), Yogyakarta, 55281 Indonesia; 4 Department of Radiology - Faculty of Medicine, Public Health and Nursing, Universitas Gadjah Mada (Dr. Sardjito Hospital), Yogyakarta, 55281 Indonesia; 5 Division of Surgical Oncology-Department of Surgery, Faculty of Medicine Universitas Andalas (Dr. M Jamil Hospital), Padang, 25127 Indonesia; 6 MRC Unit for Lifelong Health and Ageing, University College London, Place London, Bedford 33, London, UK

**Keywords:** Young breast cancer, Subtype, Progression free survival, Psychosocial need, Indonesia

## Abstract

**Background:**

Breast cancer diagnosed at a young age is often associated with aggressive biology, advanced stage, and unfavorable prognosis. The median age of breast cancer diagnosis in Indonesia is younger (48 vs. 68 years-old in Europe) with a relatively higher proportion of patients younger than 40 years old. Although prognosis and outcome of young breast cancer are well studied in developed nations, research evaluating biological characteristics, delivered treatment, and clinical outcomes is very limited in Indonesia.

**Methods:**

We analyzed all breast cancer patients who underwent surgery at Dr. Sardjito Hospital, Indonesia, in 2012–2017. Details of pathology profiles, treatment administrated, and outcomes, as well as reproductive factors among patients younger than 40 years old, were collected and analyzed. Kaplan-Meier curve was used to assess conditional survival based on baseline characteristics.

**Results:**

From the total of 1259 breast cancer patients (median age 51 years), 144 (11.4%) were younger than 40 years old (median age 37 years). Of these young patients, 19 (13.2%) were bilateral and 92 (64%) were diagnosed in advanced stages (stages IIIA-C and IV). Median tumor diameter was 5.5 cm and nodal infiltration was present in 73%. Distant metastasis was found in 16% at the time of diagnosis. Moderate and poor differentiation of tumor were 20.8 and 78.5%, respectively, and lymphovascular invasion was found in 90.3%. Around 40% were hormone receptor-positive, 30.6% human epidermal growth factor receptor 2 positive, and 38.2% triple negative. Patients underwent radical surgery in 121 cases (84%) and breast conserving surgery in 7 cases (4.9%). Adjuvant chemotherapy was administrated in 68% and hormonal therapy in 34%. Progression-free survival was significantly shorter in patients with advanced stage, skin and chest wall involvement (T4), positive lymph node infiltration, positive hormonal receptor, and triple negative subtype (log-rank Mantel-Cox tests, *p* < 0.05).

**Conclusion:**

We found a high frequency of young breast cancer with biologically more aggressive tumors, late diagnosis, frequent relapse, and poor prognosis. Further actions to improve clinical management and meet psychosocial needs in young breast cancer patients are warranted.

## Background

Breast cancer has emerged as the most diagnosed cancer and the leading cause of cancer-related mortality among women worldwide [1]. More than 2 million females are diagnosed each year with annual mortality over 600,000 [1]. In Indonesia, the total incidence of breast cancer in 2014 was ~ 50,000 and annual mortality was ~ 20,000 [2]. In general, the incidence of breast cancer in young women is relatively low worldwide. Median age at diagnosis is 68 years in developed nations [3, 4], while in Indonesia median age at diagnosis is ~ 48 years-old including more than 5000 women under 40 years who are annually diagnosed with breast cancer [2].

Young age is associated with worse prognosis in breast cancer [5, 6] which may be partly explained by different biological mechanisms in young patients. Young breast cancers (YBCs) exhibit more aggressive characteristics [7] including higher rates of mitotic index, poor tumor differentiation, triple-negative subtype, with higher propensity for relapse and distant metastasis [6, 7]. In low and middle-income countries (LMIC) including Indonesia, a delayed presentation may additionally play a role as there is a lack of population-based screening programs [8]. There is also low cancer awareness in Indonesian women, particularly those with lower household expenditure and education levels, which may contribute to a delayed diagnosis of breast cancer including among young women [8]. Specific considerations in comprehensive cancer care, surveillance plans, adherence to adjuvant therapy as well as management of side effects in YBC patients are required to achieve optimal therapeutic and long-term outcome results. However, little is known regarding the clinical characteristics associated with prognosis of young women with breast cancer in Indonesia, which could be used to better inform strategies to improve care for these patients.

To gain further understanding regarding young women with breast cancer in Indonesia, in this study we presented baseline clinicopathological factors and treatment choice and prognosis of YBC patients in a tertiary referral hospital-based case series in Indonesia. We also assessed the relationship between these factors and progression free survival (PFS) then discussed these findings in relation to other determining factors and made recommendations to improve clinical management of young patients with breast cancer in Indonesia.

## Methods

### Design and population of the study

All breast cancer patients who underwent surgery at the Department of Surgery, Sardjito Hospital in 2012–2017 were collected, which included a total of 1259 breast cancer patients. Of this population, 114 (11.4%) were 40 years old or younger, thereby meeting the definition of YBC and were included in the analysis of young patients with breast cancer. The study was approved by the Ethical Committee Faculty of Medicine Universitas Gadjah Mada Yogyakarta (1143/EC/2017) and informed consent was acquired from each participant.

### Data collection

Data were drawn retrospectively from patients’ medical records including age, clinical data, cancer stage, tumor size, lymph node infiltration, metastasis, histological grade, hormonal receptors, delivered treatments (surgery, chemotherapy, radiotherapy, and hormonal therapy) were collected and summarized.

YBC was defined as breast cancer patients aged 40 years or younger. Tumors were classified using the Tumor-Node-Metastasis (TNM) system according to the seventh edition of the^1^American Joint Committee on Cancer (AJCC) classification of 2009 [9]. Tumor grade was evaluated using the Nottingham modification of the Bloom and Richardson system (mSBR) [10]. Histological type was determined according to the World Health Organization (WHO) classification of breast cancer 2012 [11]. Vascular, lymphatic, and neural invasion were histologically quantified. Estrogen and Progesterone receptors (ER and PR) were considered positive if the nuclear expression was higher than 1% of tumor cells. Expression of human epidermal growth factor receptor 2 (HER2) was determined by immunohistochemistry (IHC) staining of cytoplasmic membrane and scored accordingly for which 0/1+ is negative and + 3 is positive. For tumors with + 2 of HER2 IHC-staining or ambiguous results, Fluorescence in Situ Hybridization (FISH) was performed and any evidence of HER2 amplification was considered as positive. Based on the ER, PR, and HER2 IHC-staining results, breast cancers were classified as Luminal A (ER+/PR+/HER2+), Luminal B (ER+/PR-/HER2+), HER2 (ER-/PR-/HER2+) or triple negative breast cancer (TNBC) (ER-/PR-/HER2-) [5].

Smoking status was assessed during interviews after diagnosis of breast cancer and was then categorized into active or former smoker if respondent actively or ever smoked more than 100 cigarettes in their lifetime. Physical activity was classified into vigorous if the activity was performed more than 2 h per week and resulted in rapid heartbeat and breathless (jogging, aerobic, rigorous swimming/cycling), moderate if the activity was performed more than 2 h per week and caused exhaustion but not breathless (easy swimming/cycling, dancing, yoga, pilates), and light activity if the activity caused tiredness but not exhaustion (walking, driving, housework). Although we did not directly measure the ratio of work to a standard resting metabolic rate (MET), the categorization of physical activity was estimated according to a specific type of activity [12]. The residence was classified into urban (*kota*) and rural (*desa*) based on official administrative status given by the Indonesian government to the place where a patient was living at the time of diagnosis (shown by their identity cards). Positive family history was defined if first- or second-degree relative had a history of breast and/or ovarian cancer. Parity was determined by the number of full-term pregnancies and categorized into null- or multi-parity.

### Follow-up

Patients received follow-up from the date of diagnosis until any tumor progression was recorded, until they died or until the last date of the study in February 2018. Follow-up visits were scheduled every month for the first 6 months and every 6 months after completion of therapy unless any non-scheduled visit was indicated, and involved a thorough clinical examination, breast sonography and/or mammography, abdominal ultrasonography, chest X-ray, and bone scan. PFS was calculated starting from the time elapsed of diagnosis to tumor progression or death from any cause.

### Statistical analysis

SPSS 17.0 software (IBM) was used for conducting the statistical analysis. Descriptive variables were presented in means ± standard deviation (SD) or medians. The Mann-Whitney-U test was used to compare continuous variables and the χ2 test to compare categorical variables. Kaplan-Meier survival curve and log-rank Mantel-Cox tests compared PFS across different clinicopathological characteristics and treatment outcomes. Cox regression was used to identify factors influencing PFS. For all comparisons, *p* < 0.05 was considered as statistically significant.

## Results

### Demographic and clinicopathological characteristics

Of the YBC patients, the median age at diagnosis was 37 years and 39.6% were younger than 35 years old (Table 1). The majority of patients were Javanese decent (and living in rural areas, 86.1%), high school graduates or of lower educational level (79%), married (97.2%), non-smokers (96.5%), and sedentary (72.9%) (Table 1). Five patients (3.5%) were diagnosed with breast cancer during pregnancies and 19 patients (13.2%) were bilateral breast cancers.

**Table 1:** Sociodemographic characteristics of young breast cancer patients (*N* = 144) and their associations with disease progression (*HR* hazard ratio, *CI* confidence interval)

Parameters	N	%	HR (95%CI)
Age at diagnosis			
Median	37		
< 35	57	39.60%	0.937 (0.376–2.273)
≥ 35	87	60.40%	ref
Ethnicity			
Javanese	130	90.30%	1.581 (0.387–6.462)
Banjar	4	2.80%	ref
Sundanese	3	2.10%	ref
Dayak	3	2.10%	ref
Chinese	2	1.40%	ref
Others	2	1.40%	ref
Residence			
Rural	124	86.10%	1.021 (0.365–2.861)
Urban	20	13.90%	ref
Education			
Less than high school	34	23.60%	0.836 (0.334–2.092)
High school	80	55.60%	ref
Higher education	30	20.80%	ref
Marital status			
Married	140	97.20%	1.985 (0.2–19.689)
Not married	4	2.80%	ref
Physical activity			
Vigorously active	17	11.80%	ref
Moderately active	22	15.30%	0.230 (0.086–0.615)
Lightly active	105	72.90%	ref
Tobacco smoking			
Never	139	96.50%	1.985 (0.2–19.689)
Ever	3	2.10%	ref
Former	2	1.40%	ref

Around 7% of patients reported having first- or second-degree relative with breast or ovarian cancer. The majority of patients had at least one full-term pregnancy (88.2%). Breast lumps and skin induration or lesions were the most common reason to seek treatment (65.3 and 25.7%, respectively). All patients reported themselves as right-handed and 47% of the tumors were located in the right breast. The median size of the tumor was 5.5 cm and the majority were larger than 3 cm (84%). Lymph node infiltration was found in 73% of cases and distant metastasis at diagnosis was discovered in 16% of cases. In total, 36% of cases were early stage-, 47.9% were locally advanced-, and 16% were metastatic breast cancers. More than 80% of YBC cases were histologically infiltrative ductal carcinomas. Positivity of hormonal receptors and HER2 expression were reported in 39.6 and 30.6%, respectively. Molecular classification using IHC-staining revealed 43% patients as luminal- and 38% as TNBC (Table 2). Ki67 IHC staining was documented in 34 patients in which 19 patients (55.8%) were above 14% indicating a high mitotic index.

**Table 2:** Clinical and pathological variables in young breast cancer patients (*N* = 144) and their associations with disease progression (*HR* hazard ratio, *CI* confidence interval)

Variables	N	%	HR (95%CI)
The family history of breast cancer			
No	133	92.40%	0.969 (0.258–3.640)
Yes	11	7.60%	ref
Parity			
Nulliparity	17	11.80%	1.925 (0.647–5.730)
Multiparity	127	88.20%	ref
Chief presentation			
Breast lump	94	65.30%	1.295 (0.6–2.795)
Skin induration or lesion	37	25.70%	ref
Axillary mass	4	2.80%	
Pain	9	6.30%	
Positive biopsy diagnosis			
Fine needle aspiration	82	56.90%	N/A
Biopsy	62	43.10%	N/A
Localization			
Right	68	47.20%	3.839 (1.045–14.112)
Left	51	39.60%	
Bilateral	19	13.20%	ref
Multifocal lumps			
No	118	82%	3.839 (1.045–14.112)
Yes	26	18%	ref
Tumor size			
Median	5.5 cm		
> 3	121	84%	3.8 (1.04–14.11)
≤ 3	23	16%	ref
Lymph node involvement			
positive	108	75%	2.159 (0.912–5.108)
negative	36	25.00%	ref
Stage			
I	5	3.50%	ref
II	47	32.60%	
III	69	47.900%	0.610 (0.278–1.339)
IV	23	16%	
Distant metastasis at diagnosis			
Yes	23	16%	8.627 (1.909–38.994)
No	121	84%	ref
Histology			
Ductal	117	81.30%	1.568 (0.289–8.501)
Lobular	12	8.30%	ref
Ductolobuler	8	5.60%	
Others	7	4.80%	
mSBR grading			
I	1	0.70%	0.836 (0.334–2.092)
II	30	20.80%	
III	113	78.50%	ref
Angioinvasion			
No	6	4.20%	0.761 (0.134–4.335)
Yes	138	95.80%	ref
Lymphatic invasion			
No	14	9.70%	2.513 (0.668–9.447)
Yes	130	90.30%	ref
Hormonal receptor			
Negative	86	59.70%	1.6 (0.753–3.398)
Positive	57	39.60%	ref
HER2 expression			
Negative	99	68.80%	0.623 (0.281–1.382)
Positive	44	30.60%	ref
Molecular classification			
Triple Negative	55	38.20%	2.209 (0.945–5.162)
Luminal	62	43.10%	ref
HER2	26	18.10%	

### Treatment choice

Operable breast cancers (T1-T2 N0-N1 M0 and T3N0M0) were diagnosed in 36% (*N* = 52) and 13.5% of the patients who received breast-conserving surgery (Table 2 and Table 3). Locally advanced breast cancers (N2N3M0, T4 M0, and T3N1M0) represented nearly 50% of cases (*N* = 69) and 8.7% of those who received neoadjuvant chemotherapy. In total, radical and palliative surgery was performed in 84 and 11% of cases, respectively. Adjuvant chemotherapy was completed in 68% of cases meanwhile nearly 12% of patients did not receive chemotherapy. Of 57 patients with hormone receptor positive, 86% received hormonal therapy. Targeted therapy in HER2-positive tumors was delivered in 41% of cases (18 of 44 patients). In addition, bisphosphonate treatment was administrated in 74% of patients with bone metastasis. Around 18 and 20% of patients did not show up for chemotherapy and radiotherapy, respectively. Comparing patients who received and did not receive chemotherapy (118 vs. 26 patients) and radiotherapy (102 vs. 41 patients) with their education levels (less or higher than high school, 34 vs. 110 patients) and residence (rural-urban, 124 vs. 20 patients) resulted in no statistical difference. As a result, barriers to seeking chemotherapy and radiotherapy were not associated with residence and education levels (Fisher exact test, *p* > 0.05).

**Table 3:** Treatment modalities in young breast cancer patients (*N* = 144) and their associations with disease progression (*HR* hazard ratio, *CI* confidence interval)

Treatment modalities	N	%	HR (95%CI)
Surgery			
Palliative	16	11.10%	2.915 (0.775–10.965)
Radical	121	84%	ref
Conservative	7	4.90%	
Chemotherapy			
Neoadjuvant	6	4.20%	1.667 (0.310–8.976)
Adjuvant	98	68%	
Palliative	23	16%	
No	26	11.80%	ref
Radiotherapy			
No	29	20.10%	0.836 (0.321–2.1770
Waiting list	13	9%	
Yes	102	70.80%	ref
Hormonal therapy			
Yes	49	34%	ref
No	8	5.60%	1.04 (0.49–2.207)
HR-negative	86	59.700%	
Anti-HER2 therapy			
HER2 positive	44	30.60%	
No	26	18.10%	0.844 (0.203–3.504)
Yes	18	12.50%	ref
Bisphosphonate treatment			
Yes	17	11.80%	N/A
No	6	4.20%	N/A
Bone metastases	23	16%	

### Progression-free survival (PFS) data

**Fig. 1:**
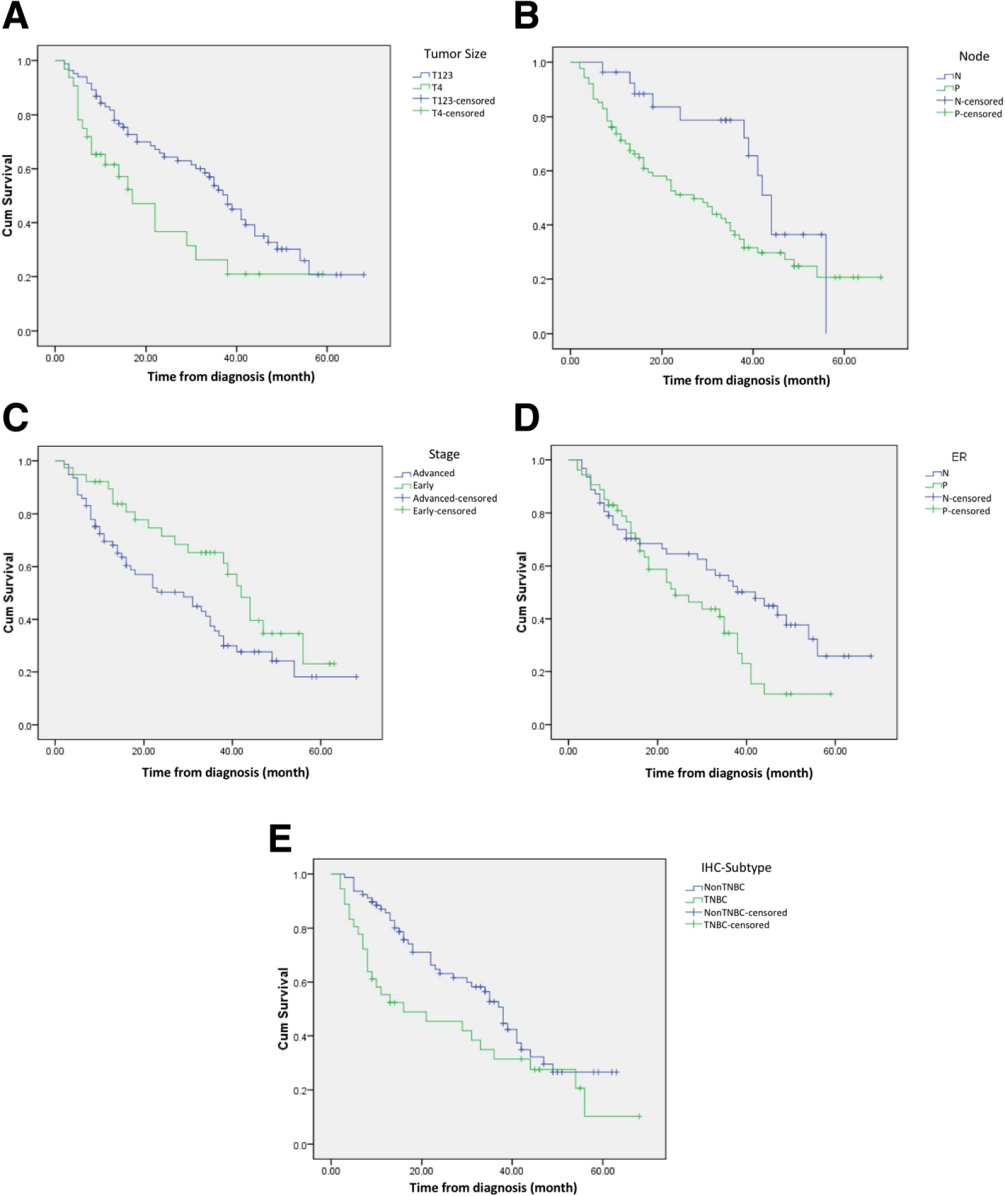
Progression free survival correlated to some parameters in YBCs. PFS of YBCs was significantly shorter (**a**) in YBCs with skin or chest wall infiltration (T4) compared to those without skin/chest wall infiltration (T1-T3) (median survival 16 vs. 35 months, *p* = 0.011; HR = 1.89, 95%CI: 1.12–3.21); **b** in YBCs with positive lymph node infiltration compared to node negative (median survival 24 vs 42 months, *p* = 0.041; HR = 1.95, 95%CI: 1.05–3.64); **c** in YBC with hormonal receptor positive compared to negative (median survival 42 vs. 29 months, *p* = 0.37; HR = 1.64, 95%CI: 1.01–2.67), and (**d**) in triple-negative YBCs compared to other subtypes (median survival 16 vs 38 months, *p* = 0.038; HR = 1.66, 95%CI: 1.02–2.72)

Twenty-nine patients were lost during follow-up, so PFS data were only available for 115 (80%) patients. Therefore, interpretations related to PFS need to be considered according to the context with potential surveillance or selection bias on the reported prevalence of relatively longer survival of YBC patients. During follow-up, 35 (24.3%) patients developed distant metastasis and 10 (6.9%) suffered from locoregional recurrence after a mean duration of 20.3 months. Survival probabilities based on clinicopathological factors are shown using the Kaplan-Meier curve in Fig. 1. Based on log-rank Mantle-Cox tests, PFS was significantly higher in early stages (stages I-II) compared to more advanced stages (median survival 42 vs. 29 months respectively, *p* = 0.037; HR = 0.58, 95%CI: 0.34–0.98). Shorter PFS was found in patients with skin and chest wall infiltration (T4) compared to those without skin/chest wall infiltration (T1-T3) (median survival 16 vs. 35 months, *p* = 0.011; HR = 1.89, 95%CI: 1.12–3.21), positive lymph node infiltration (median survival 24 vs. 42 months, *p* = 0.041; HR = 1.95, 95%CI: 1.05–3.64), hormonal receptor-positive compared to negative (median survival 42 vs. 29 months, *p* = 0.37; HR = 1.64, 95%CI: 1.01–2.67), and triple-negative breast cancer (TNBC) compared to other subtypes (median survival 16 vs. 38 months, *p* = 0.038; HR = 1.66, 95%CI: 1.02–2.72), (Fig. 1).

## Discussion

The proportion of YBCs in our study (11.4%) was twice as high as the frequency in the US and Europe (5–7%) [13, 14]. This finding supports earlier data suggesting lower age at diagnosis in Indonesia than those countries [2]. The reason for these differences is not clear. Biologically, YBCs in our study showed aggressive phenotypes with relatively high proportion of large tumor size, lymph node involvement, advanced and metastatic diseases, high histology grade, angiolymphatic invasion, Her-2 positivity, TNBC subtype, as well as frequent progression and relapse during follow-up (Table 2) in accordance with previous studies [6, 15–17]. In a large prospective study (*N* = 2956) investigating factors related to poor prognosis in YBCs, 50% subjects were lymph node positive, 30% were hormone receptor negative, 20% patients were TNBC subtype, and 60% had histologically poor differentiation [18].

Breast-conserving surgery (BCS) and sentinel node biopsy followed by breast radiation are recommended for YBCs [19]. Only 5% of our patient cohorts were assigned for BCS for the reason of high proportion of advanced stages and the long waiting list for radiotherapy. In addition, cosmetic consideration, immediate breast reconstruction surgery, and impact on body image and sexuality should be offered to YBCs [19]. In advanced cancer, however, radical surgery is preferred to achieve locoregional control and reduce relapse.

Around 88 and 70% of YBCs in this study completed chemotherapy and radiotherapy, respectively. Hormonal therapy was delivered in 84% of patients with hormone receptor positive. No specific chemotherapeutic regiment is recommended for YBCs and regiments are usually assigned according to IHC-subtyping [19]. Recent trials using multigene assays suggest that ER-positive and premenopausal early-stage breast cancer (node negative) with low recurrence score might not require chemotherapy [20, 21]. The trials included YBCs (3.5 and 2% in TAILORx and MINDACT, respectively) [20, 21] although application of multigene tests is still far from a practice routine in LMICs including Indonesia. However, the most important challenge in Indonesia is to diagnose young women in the early stages (T1-T2, node negative). Hormonal therapy is recommended in premenopausal women with hormonal-receptor positive (≥1% cell positive for ER or PR) although patients with ≥10% cell positive are the most responsive to adjuvant hormonal therapy [22]. Radiotherapy with the addition of regional nodal irradiation (RNI) is correlated with better PFS and stronger effect is found in patients with ER-negative which is more common in YBCs [23]. Despite following general recommendations for treatment of young breast cancer, the frequency of disease progression in our cohort is relatively high (24%). We also found a high proportion of patients who did not receive chemotherapy and radiotherapy. Factors associated with barriers to treatment (surgery, chemotherapy, and radiation) as well as to therapy adherence in YBCs require further study [24]. In addition to the aggressive biology of YBCs, advanced stages at diagnosis contribute to the frequent relapse and unfavorable PFS (Fig. 1 section A). The majority of YBC patients were living in a rural residence with low education levels which might contribute to the late presentation for medical treatment although our study indicated that those factors were not associated with barriers to receive chemotherapy and radiotherapy. Our previous study showed relatively low breast cancer awareness among Indonesian women particularly those with low social economic status and low education levels [8]. Misunderstanding about breast cancer and the benefits of early detection might also hamper health-seeking attitudes.

More than 13% of patients in our study developed bilateral breast cancer and 7.6% with positive family history implicating the increased genetic risk in YBCs. However, genetic service, in general, is not available in most LMICs including in Indonesia. One previous study suggested that genetic counseling and understanding of personal genetic risk is associated with distress reduction and improvement of patient’s wellbeing [25]. YBC patients also need particular attention regarding psychosocial factors and fertility concern [26, 27]which were not yet adequately addressed in our clinics. Identification of unmet psychosocial needs and appropriate interventions are important to promote psychological acceptance to the disease [26, 28, 29]. In addition, advanced cancers also are associated with increasing cancer-related disability and poor functioning which require rehabilitation to improve quality of life. However, we previously reported that cancer rehabilitation is also either not available or sub-optimally delivered in developing countries [30, 31].

This present study appears as the first in a series focusing on the clinicopathological profile, treatment choice, and prognosis in Indonesian YBCs. A limitation in this study was that we only included breast cancer patients who were treated in the Department of Surgery, thereby our data may have not included terminally ill patients for whom any surgery was not indicated. This may have resulted in an underestimation of disease progression in our study. A population-based study is, therefore, necessary to replicate our findings in a wider population. Additionally, the hospital is a tertiary referral hospital that treats more complex and advanced stage cancers because a vertical referral system has been implemented in the national health insurance [32]. More collaborative studies are required since the nation-wide cancer registry in Indonesia is not yet comprehensively available [33] to record all YBCs, demographic data, pathological profiles, and survival rates.

## Conclusions

There was a higher proportion of YBC compared to the rates in developed countries. Identification of both biological and clinical factors associated with effective treatment and prognosis are essential to improve YBC patients’ outcome and well-being. Several challenges in managing YBCs in Indonesia are a late presentation at diagnosis, frequent relapse and poor survivals, and aggressive type of cancer, with high rates of non-adherence to adjuvant chemotherapy and radiotherapy which may be addressed through further research.

## Abbreviations

AJCC: American Joint Committee on Cancer
BCS: Breast Conserving Surgery
BMC: BioMed Central
CI: Confidence Interval
ER: Estrogen Receptor
FISH: Fluorescence in Situ Hybridization
HER2: Human epidermal growth factor receptor 2
HR: Hazard Ratio
IBM: International Business Machine Corporation
IHC: Immunohistochemistry
LMIC: Low-Middle Income Countries
MINDACT: Microarray in Node-Negative and 1 to 3 Positive Lymph Node Disease May Avoid Chemotherapy
mSBR: Modified System Bloom and Richardson
PFS: Progression Free Survival
PR: Progesterone Receptor
RNI: Regional Nodal Irradiation
SPSS: Statistical Package for the Social Sciences
TAILORx: *Trial* Assigning Individualized Options for Treatment
TNBC: Triple-Negative Breast Cancer
TNM: Tumor Node Metastasis
WHO: World Health Organization
YBC: Young Breast Cancer

## Footnotes

1 mSBR is modified Scarff Bloom Richardson grading system (also known as Nottingham system) in which tumor is semi-quantitatively assessed according to percentage of tubule formation (score 1–3 for 75%, 10–75, and < 10%), nuclear polymorphism (score 1–3 for uniform small cells, moderate variation, marked variation), and mitotic counts per 10–40 fields (score 1–3 for 0–5, 6–10, and > 11). The sum of the score determines the grade: Grade 1 [3–5], Grade 2 [6, 7], Grade 3 [8, 9]. (REF #10)
